# Contribution to the Study of the Differences between Kreosote and Carbolic Acid

**Published:** 1868-04

**Authors:** S. C. Bensow

**Affiliations:** Royal Court Dentist of Stockholm.


### ARTICLE V.

### *Contribution to the Study of the differences between Kreosote and Carbolic Acid.*

By S. C. Bensow, Royal Court Dentist of Stockholm.

Translated from the German Quarterly Journal of Dental Medicine.

As early as 1853, Gorup-Besanez showed that the genuine kreosote of Reichenbach is wholly different from any other, as Carbolic or Phenic acid, which is also a product of the dry distillation of organic matter and which is met with in greatest abundance in coal-tar. In smell and in many other physical and chemical properties, these products are very much alike, especially when impure, and several chemists regarded phenic acid as the principal and characteristic ingredient of kreosote, and set down the word kreosote, among other appellations as a synonym of phenic or carbolic acid.

The more or less pure carbolic acid obtained from coal-tar is what is ordinarily sold for kreosote in the shops, and that for the sole reason that the rates of the medical taxes are founded upon the Druggists' price-current for kreosote, but without specifying that it shall be Reichenbach's or beech-tar kreosote which is also quoted in the Price-Current but at a much higher price. Down to our own times many doubted that any real difference existed between these two.

A. E. Hoffmann made a new examination of Reichenbach's kreosote (Journal of Practical Chemistry, 1865, p. 225,) and arrived at conclusions totally opposed to those of Gorup-Besanez and several others, saying that this kreosote is nothing but an impure phenic acid. This prompted Gorup-Besanez to a renewed investigation of a kreosote, of the purity of which he was fully convinced, viz., that from the Chemical Industrial Association in Mainz. This, in all essentials, behaved precisely as that formerly examined which he got from Blansko, a factory in Mahren now discontinued, which leads him to doubt the purity of the kreosote with which Hoffmann worked. The essential difference of Reichenbach's kreosote from carbolic or phenic acid is also established by the researches of Volkel, Hlasiwetz and H. Muller.*

The Rhenish kreosote from Mainz, though closely resem-

---

*Ann. d. Ch. u. Ph., LXXVIII. p. 231; LXXXVI. p. 66 and 223; XCVI. p. 39: CII. p. 145; CVI. p. 339; Zeitschr. f. Ph. Ch. 1464, p. 703.

bling the Bohemian, and that of Mahren, is not absolutely identical with them. Subjected to a protracted digestion over a slow fire with carbonate of potash and muriatic acid, till chlorine is plentifully evolved, and it is converted into a hard, plaster-like mass after cooling, filled with gold-yellow, glittering, crystalline scales ; and these crystalline bodies purified by recrystalligation from alcohol, and still further by sublimation after pressure between folds of blotting-paper, it yields, as does also the kreosote from Mahren, a hexachlorxylon, which, as may be observed in the sublimation, is a mixture of two distinct compounds. These can be separated by chloroform, one of them, $C_{16}\ H_4\ Cl_4\ O$ being soluble, and the other $C_{14}\ H_2\ Cl_4\ O_4$ insoluble in cold chloroform. In the product from Rhenish kreosote, the last is the principal component. That from the kreosote of Mahren has for its chief ingredient $C_{16}\ H_4\ Cl_4\ O_4$ and a mixture of two others, $C_{16}\ H_5\ Cl_3\ O_4$ and $C_{14}\ H_3\ Cl_3\ O_4$. The first named compounds are homologous with chlorinetted quinone and their formation from kreosote may be comprehended by the following formulæ :

$$C_{14}\ H_8\ O_4 + 10\ Cl = C_{14}\ H_2\ Cl_4\ O_4 + 6\ H\ Cl$$
$$C_{16}\ H_{10}\ O_4 + 10\ Cl = C_{16}\ H_4\ Cl_4\ O_4 + 6\ H Cl$$

The Rhenish kreosote, therefore contains the same ingredients as guiacol* (the heavy kreosote-like oil, soluble in potash-ley, obtained from the products of the dry distillation of guiacum resin) which, according to the researches of Hlasiwetz may be regarded chiefly as a mixture of $C_{14}\ H_8\ O_4$ and $C_{16}\ H_{10}\ 0^4$, so that one is compelled to ask himself if the two products kreosote and guiacol are not identical. According to a communication from H. Muller to Gorup-Besanez, English kreosote, prepared from Swedish tar, consists mainly of $O_{16}\ H_{10}\ O_4$. The $C_{16}\ H_{10}\ O_4$ of guiacol forms with potash $C_{16}\ H_9\ KO_4$. This compound is obtained when kreosote is briskly agitated with moderately strong

*This appears to be the hydride of guiacyl of the French chemists, who give the formula $C_{14}\ H_8\ O_4$. TR.

ammonia, rectified, poured into its own volume of ether, and then treated with a slight excess of a concentrated solution of potash in alcohol.

Dr. K. Frisch had also undertaken an investigation of the pure kreosote from Mainz, in consequence of the different results of Hoffmann on the one side and Gorup Besanez and others on the opposite side. The kreosote was light brown, almost colorless, of specific gravity 1.0874 at 20°, incapable of crystallization till reduced to 16°. Upon distillation, the larger portion of it went over at above 204°. Elementary analysis gave results which correspond to Volkel's empirical formula for kreasote, $C_{24}\ H_{14}\ 0_5$. Its principal component is kreosol, $C_{16}\ H_{10}\ 0_4$. Upon treating it with a concentrated solution of potash in alcohol, a large quantity of kreosolate of potash ($C_{16}\ H_9\ KO_4$) is obtained. The second component of the kreosote cannot be obtained from the mother-liquor of this salt, but must be sought for by other and indirect means.

If kreosote is added drop by drop to concentrated nitric acid, there is formed, together with oxalic acid, a yellow resin-like substance, which after recrystallization from alcohol is obtained in beautiful acicular crystals, and is nothing else than picric (trinitrophenic acid.) This must come from the second component of kreosote, as kreosote gives no crystalline compound with nitric acid. If kreosote is digested with sulphuric acid, the solution diluted with water and boiled with nitric acid, there is obtained, together with oxalic acid, a second nitrogenous compound, which by its properties and by elementary analysis, shows itself to be a dinitrophenic acid. It separates as a crystalline resin, which when dissolved in ammonia and the solution decomposed by nitric acid, is obtained in prismatic crystals. Oxalic acid, according to Hlasiwets, originates from kreosol, but the nitrocompounds must arise from a phenyl combination. Gorup Besanez's Hexachlorxylon too, he found to consist of two chlorine compounds closely resembling one another in external appearances. He obtained from them chloranilic acid with several compounds of chlorine and quinine, and believes that hexchlorxylon is a mixture from Tetrachlorhydroquinone. By the continued chlorination of kreosote he obtained chloranil ($C_{12}$ $Cl_4$ $O_4$), so that this also proves that the second body discovered in kreosote besides kreosol, is a phenyl compound, a view confirmed by the following experiment. Dissolve kreosote in concentrated sulphuric acid and let the solution stand 24 hours at a temperature of 50°, so that when diluted with water there is no precipitate, but only a clear dark ruby-red liquid, which is decolorized by zinc. Upon neutralizing this red liquid with carbonate of baryta, filtering and evaporating the filtrate in vacuo over sulphuric acid, the sulphophenylate of baryta described by Laurent is obtained ($C_{12}$ $H_6$ O $SO_3$ + HO) + (BaO $SO_3$). The corresponding lead salt may also be obtained.

It is probable that there are several different kinds of kreosote which are mixed together, and the purified kreosote becomes richer in acid through the action of potash lye in the process of rectification. He thinks that his researches justify the assertion that kreosote may be regarded as a union of kreosol with a phenyl compound. His opinion is that kreosote in its composition resembles acid kreosolate of potash in which the potassium is represented by phenyl.

$$C_{16}\,H_{10}\,O_4\;C_{16}\,H_9\,(\,C_{12}\,H_5\,)\,O_4 + HO.$$

In an alcoholic solution of chloride of iron, pure kreosote gives a green tint, phenylic acid a brown. In a watery solution kreosote has no reaction, while phenylic acid gives the well-known blue color.

With three or four volumes of a saturated baryta water kreosote gives only an incomplete cloudy solution. Phenylic acid gives a clear solution which after standing deposits nothing or only a slight pulverulent sediment.

In liquor ammoniæ, at common temperatures, neither kreosote nor phenylic acid dissolves. On warming, kreosote still refuses to dissolve and after shaking settles out again with a beautiful yellow tint. Phenylic acid forms a clear solution with warm aqua ammoniæ, but separates after cooling with a violet brown discoloration. In dilute potash ley both dissolve but kreosote with difficulty. Kreosote requires much ley for complete solution, otherwise a portion of the kreosote settles out during agitation as the solution cools ; addition of ley clears up the solution. Phenylic acid gives a clear and permanent solution with little ley.

To two drops of liquor ammoniæ in a test tube add a solution of chloride of iron of such strength that the precipitate is redissolved on the addition of about four volumes of water. When a few drops of kreosote are added, the solution becomes first green, then brown. Phenylic acid gives a blue or violet tint

Collodion gives with its own volume of kreosote a stiff somewhat opalescent jelly, but with phenylic acid, a viscous (dick fliessende) clear solution.

---

